# Conformal radiotherapy for lung cancer: interobservers' variability in the definition of gross tumor volume between radiologists and radiotherapists

**DOI:** 10.1186/1748-717X-4-28

**Published:** 2009-08-05

**Authors:** Chiang J Tyng, Rubens Chojniak, Paula NV Pinto, Marcelle A Borba, Almir GV Bitencourt, Ricardo C Fogaroli, Douglas G Castro, Paulo E Novaes

**Affiliations:** 1 Department of Diagnostic Imaging, Hospital A C Camargo, Rua Prof. Antônio Prudente, 211, São Paulo - SP, Brazil; 2 Department of Radiotheraphy, Hospital A C Camargo, Rua Prof. Antônio Prudente, 211, São Paulo - SP, Brazil

## Abstract

**Background:**

Conformal external radiotherapy aims to improve tumor control by boosting tumor dose, reducing morbidity and sparing healthy tissues. To meet this objective careful visualization of the tumor and adjacent areas is required. However, one of the major issues to be solved in this context is the volumetric definition of the targets. This study proposes to compare the gross volume of lung tumors as delineated by specialized radiologists and radiotherapists of a cancer center.

**Methods:**

Chest CT scans of a total of 23 patients all with non-small cell lung cancer, not submitted to surgery, eligible and referred to conformal radiotherapy on the Hospital A. C. Camargo (São Paulo, Brazil), during the year 2004 were analyzed. All cases were delineated by 2 radiologists and 2 radiotherapists. Only the gross tumor volume and the enlarged lymph nodes were delineated. As such, four gross tumor volumes were achieved for each one of the 23 patients.

**Results:**

There was a significant positive correlation between the 2 measurements (among the radiotherapists, radiologists and intra-class) and there was randomness in the distribution of data within the constructed confidence interval.

**Conclusion:**

There were no significant differences in the definition of gross tumor volume between radiologists and radiotherapists.

## Background

Lung cancer is becoming increasingly frequent in both genders worldwide. Three-dimensional conformal radiotherapy has been utilized for non-small-cell lung cancer, especially for those in advanced stage or for the inoperable early-stage diseases. Conformal external radiotherapy is based on the extensive use of modern medical imaging techniques, efficient dosimetric software, accurate patient positioning methods, stringent verification and quality control of procedures, aiming to increase tumor control by boosting tumor dose, reducing morbidity and sparing healthy tissues. Refined visualization of the tumor and adjacent areas is required to attain this objective.

Computerized planning has to calculate with accuracy and show the dose throughout the irradiated volume of the patient, taking into account the shape of the field and the modification devices of the beams used to obtain a conformal and homogeneous dose in the target volume. The idea of giving shape to the radiation fields, in order to shape only the target volume, is referred to as "target-driven planning" and is the primary difference between conformal (3D) and conventional (2D) radiotherapy. Conformal radiotherapy permits better adaptation of the dosimetric distribution to the tumor volume, reduction of healthy organs exposure, and on the long term, higher dose of tumor irradiation [1-5].

The volume of the tumor mass (gross tumor volume) represents the area of greatest concentration of tumor cells. It is usually defined as the tumor clinically evident and visible in imaging studies, such as computed tomography or magnetic resonance. The appropriate use of the imaging study is crucial upon definition of tumor volume.

In the majority of cases, toxicities of degrees 3 to 5 are lower than 10% in patients tested with higher doses, using three-dimensional conformal radiation therapy techniques [6-8].

However, one of the most difficult problems to solve in this context is the volumetric definition of targets [5,9,10]. The high precision of this radiotherapic technique demands a stringent and qualified approach by means of therapeutic preparation procedures [11,12]. Methodological rules should be established for volumetric definition of targets, taking into account the difficulties in delineating the macroscopic volume of the target and its microscopic involvement [5,13-15].

Delineation is generally performed in many centers by radiotherapists who often have no training or experience in radiology, making it harder to accurately identify the details of anatomic structures in computed tomography imaging. With the more generalized use of conformal radiotherapy and other new technologies, the immediate need of assuring the quality control in the definition of gross tumor volume was evidenced [16].

On the other hand, although radiologists are better qualified to interpret radiological anatomy, they are not always familiar with the natural history of the disease. Differences in delineation can, therefore, be observed among physicians due to imprecise tomographic data or divergent planning. These differences have already been reported in literature for delineation of prostate, lungs, central nervous system or esophagus tumors [9,17-23], but the magnitude of all these differences is still not completely assessed.

The objective of this study is to compare the delineation of gross tumor volume of lung tumors among experienced radiologists and radiotherapists from an oncology reference center on Brazil.

## Methods

Chest CT scans of all the patients with non-small-cell lung cancer, not submitted to surgery and referred to conformal radiotherapy of Hospital A. C. Camargo (São Paulo, Brazil) during the year 2004 were analyzed.

All the tomographic exams were performed in the adequate position for treatment in the same tomography equipment (GE HiSPEED), with identical acquisition parameters and injection of endovenous contrast medium. Each acquisition was carried out in patients with apnea, in the helicoidal mode, with pitch of 1 and slice thickness of 7 mm reconstructed every 5 mm.

A total group of 23 patients was analyzed, of which 9 were females and 14 males. The average age was 69 years, ranging from 53 to 85 years. At the time of the diagnosis, 5 were in clinical stage IB; 5, in IIB; 6, in IIIA; 6, in IIIB; and 1, in IV.

The 23 cases were delineated by two radiologists and two radiotherapists from Hospital A. C. Camargo.

Each physician has received a written summary of the medical records of each patient. Only the gross tumor volume (i.e., the visible primary tumor and the enlarged lymph nodes) was delineated. According to definitions of the International Commission on Radiation Units and Measurements-ICRU (1993, 1999) the gross tumor volume is the visible or palpable tumor extension. As regards lymph nodes, those whose smaller axis diameter is larger than or equal to 1 cm are considered compromised. The lymph nodes were included in the delineation of the gross tumor volume, when located close to the primary tumor, or were delineated separately, if distant. We analyzed the gross tumor volume as a whole: both the primary tumor and the enlarged lymph nodes in each section. The optimal visualization parameters were defined in a prior study, with -600/1600 UH for the pulmonary window and +20/400 for the mediastinal window considered mandatory for delineation [24]. The magnification factor was chosen by the physician. The previous delineation was recorded, but was not made available to the other physicians. For gross tumor volume calculation, delineation was performed with the ECLIPSE^® ^software from VARIAN with the electronic cursor in each tomographic section, being thus the tumor area multiplied by the slice thickness, and the total volume resulted from the sum of the tumor volume of all slices. In this manner, we obtained 4 gross tumor volumes for each one of the 23 patients.

The measurements were initially analyzed descriptively by means of the averages calculation, as well as the standard deviations and medians and the observation of minimum and maximum values.

The statistical methods utilized were Pearson's correlation coefficient, the Bland-Altman plot, the intraclass correlation coefficient described by Fleiss and the coefficient of variation. The level of significance utilized for the tests was 5%.

## Results

Table 1 shows the average, standard deviation, median, minimum and maximum values observed by the radiologists and radiotherapists.

**Table 1:** Values of average, standard deviation, median, minimum and maximum of the values observed by the radiologists and radiotherapists

**Observer**	**Average**	**SD**	**Median**	**Minimum**	**Maximum**
Radiotherapist 1	140.84	136.29	83.56	13.03	516.85
Radiotherapist 2	137.74	141.68	78.81	11.22	496.39
Average	139.29	138.61	74.44	12.13	496.26
					
Radiologist 1	127.13	128.03	72.36	13.87	450.26
Radiologist 2	158.48	169.21	65.36	12.09	547.91
Average	142.80	141.24	68.27	12.98	465.35

**Figure 1:**
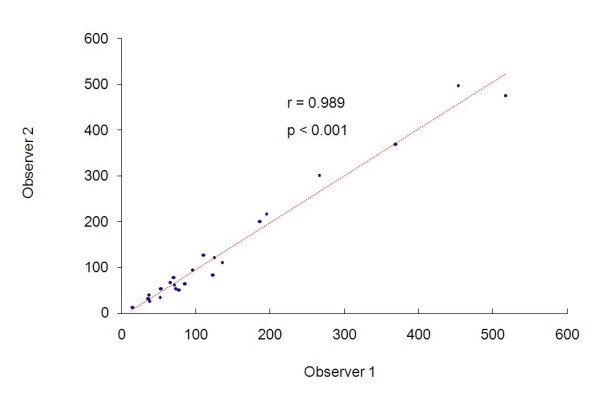
**Measurements of the radiotherapists and the value of Pearson's correlation coefficient**.

Analyzing the measurements of the radiotherapists, we can see represented in figure 1, the two measurements of the radiotherapists and the value of Pearson's correlation coefficient, in which we observe significant and positive correlation between the two measurements. The intraclass correlation coefficient for the radiotherapist is 0.989 (p < 0.001) with confidence interval of 95% equal to (0.974; 0.995).

**Figure 2:**
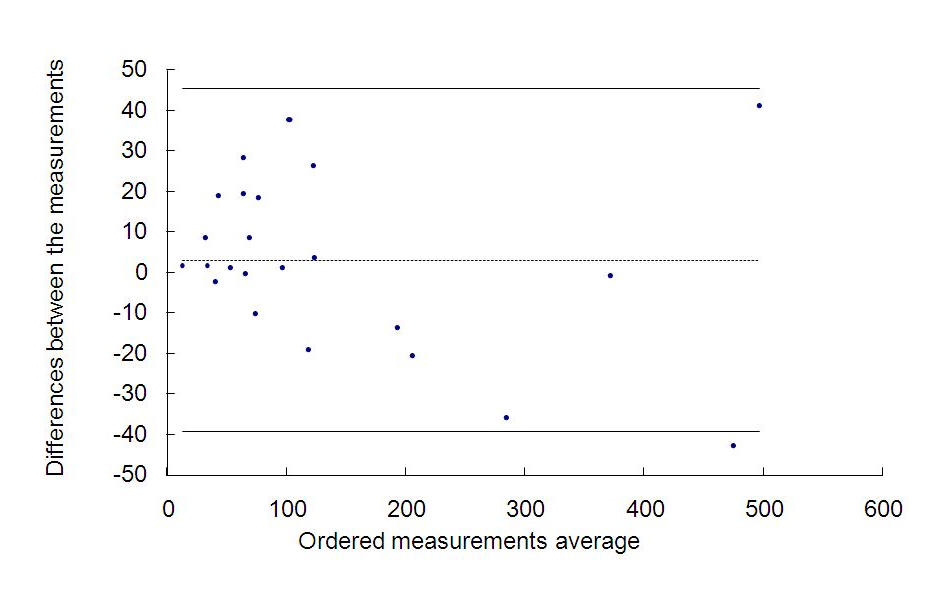
**Measurements of the radiotherapists and the graph by the Bland-Altman method**.

We can also evaluate this concordance by the Bland-Altman method. The graph representing this analysis is showed in Figure 2. The differences between the measurements ranged from - 42.88 to 37.74, with average of 3.10 and standard deviation of 21.15. Thus we obtained a confidence interval of 95% equal to (-39.20; 45.41).

**Figure 3:**
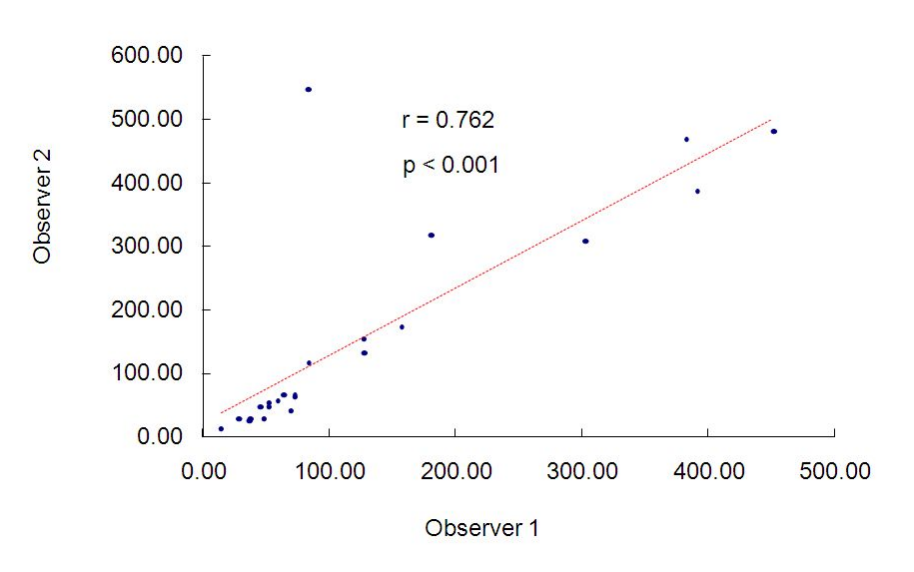
**Measurements of the radiologists and the value of Pearson's correlation coefficients**.

Analyzing the radiologists' findings, Figure 3 shows the measurements they attained and the value of Pearson's correlation coefficient, in which we observe significant and positive correlation between the two measurements. The intraclass correlation coefficient is equal to 0.762 (p < 0.001) with confidence interval of 95% equal to (0.522; 0.891).

**Figure 4:**
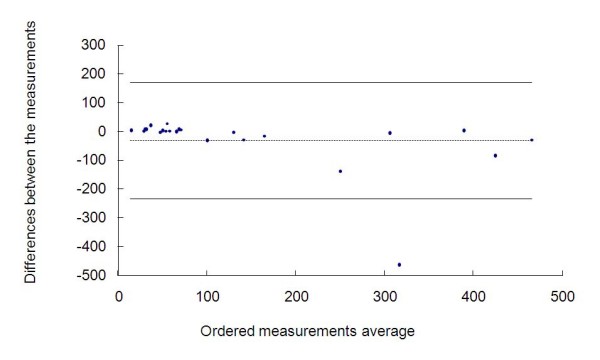
**Measurements of the radiologists and the graph by the Bland-Altman method**.

We can also evaluate this congruity by the Bland-Altman method. Figure 4 shows the graph representing this analysis. The differences between the measurements ranged from -466.27 to 26.23 with average of -31.35 and standard deviation of 101.27, thus we obtained a confidence interval of 95% equal to (-233.88; 171.18).

Analyzing radiotherapists and radiologists findings, we utilized the average between the measurements of the radiologists and the average of the measurements of the radiotherapists.

**Figure 5:**
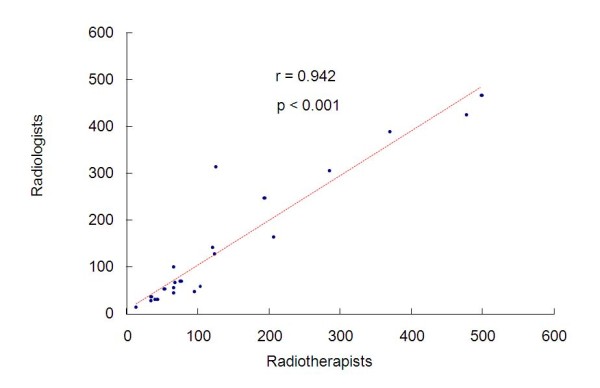
**Measurements of the radiotherapists and the radiologists and the value of Pearson's correlation coefficient**.

Figure 5 represents the measurements of the radiologists and of the radiotherapists and the value of Pearson's correlation coefficient, in which we observe significant and positive correlation between the two measurements. The intraclass correlation coefficient is equal to 0.942 (p < 0.001) with confidence interval of 95% equal to (0.869; 0.975). Hence an excellent correlation between the two measurements has been found.

**Figure 6:**
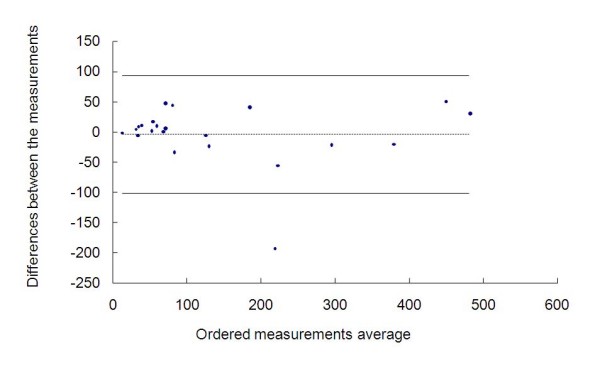
**Measurements of the radiotherapists and the radiologists and the graph by the Bland-Altman method**.

We can also evaluate this congruity by the Bland-Altman method. The graph representing this analysis is contained in figure 6. The differences between the measurements ranged from -192.09 to 50.14 with average of -3.51 and standard deviation of 48.73, thus we obtained a confidence interval of 95% equal to (-100.98; 93.95).

In table 2, we calculate the coefficient of variation among the 4 measurements, to wit: those of the 2 radiotherapists and those of the 2 radiologists, which once again indicates good congruity among them, with the exception of only one value.

**Table 2:** Coefficient of variation (COV) among the 4 measurements (2 radiologists and 2 radiotherapists)

**Patient**	**Radiotherapist 1**	**Radiotherapist 2**	**Radiologist 1**	**Radiologist 2**	**Average**	**COV**
1	108.55	127.42	127.29	155.28	129.64	0.15
2	369.29	370.13	391.36	387.46	379.56	0.03
3	125.05	121.29	125.97	130.56	125.72	0.03
4	13.03	11.22	13.87	12.09	12.55	0.09
5	52.13	33.13	35.87	27.78	37.23	0.28
6	68.45	78.81	72.54	65.36	71.29	0.08
7	83.56	65.31	72.36	64.18	71.35	0.12
8	35.90	27.40	46.20	27.02	34.13	0.26
9	77.58	49.27	67.62	41.39	58.97	0.28
10	135.91	109.46	81.64	547.91	218.73	**1.01**
11	65.30	65.82	83.64	116.94	82.93	0.29
12	186.47	199.94	178.92	317.77	220.78	0.30
13	453.51	496.39	382.78	466.78	449.87	0.11
14	33.94	32.31	28.72	28.98	30.99	0.08
15	195.41	215.96	157.15	172.20	185.18	0.14
16	71.31	62.83	64.20	67.13	66.37	0.06
17	53.26	52.13	52.19	51.76	52.34	0.01
18	516.85	475.67	450.26	480.44	480.81	0.06
19	266.67	302.54	303.24	308.28	295.18	0.06
20	95.41	94.10	50.23	46.40	71.54	0.38
21	121.23	83.49	59.19	56.56	80.12	0.37
22	73.29	53.71	43.71	47.11	54.46	0.24
23	37.24	39.65	34.97	25.58	34.36	0.18

## Discussion

Inoperable lung cancer prognosis remains very poor. Besides the alternate fractionated schemes and combined therapies, new planning strategies, including conformal radiotherapy and dose increase, are under investigation [25-29].

It is known that the general survival rate, cause-specific survival and local tumor control are directly correlated with the gross tumor volume in cm3. In the multivariate analysis the most predictive independent survival variable is the gross tumor volume [30].

Recently, the data mentioned by LEUNENS et al. (1993), evidenced that the gross tumor volume definition is not that simple, and there can be risks in excessive confidence in the medical capacity to estimate the tumor extension with the imaging approaches [17].

Due to the number of uncertainties and of phenomena related to the tumor, the definition of gross tumor volume in thoracic radiotherapy could result in greater volume variations [22,27,31,32], focused on the definition of lung cancer gross tumor volume as part of a delineation protocol. The three authors concluded that there is significant variation in target volume definition.

VAN DE STEENE et al. (2002), showed unexpected major interobservers' variability, with tumor delineation varying by several centimeters, due to:

1) difficulty in discriminating between tumor and atelectasia;

2) difficulty in distinguishing normal and pathological structures of the tumor;

3) use of different tomographic windows and partial volume effects;

4) insufficient anatomic knowledge [33].

Another study carried out by GIRAUD et al. (2002) that compared the delineation of gross tumor volume performed by radiologists and radiotherapists, showed significant differences between the two groups: radiologists tended to delineate lower and more homogeneous volumes than radiotherapists, especially in the "difficult" cases [34]. The delineation of the target volume and high-risk organ constitutes a critical stage in conformal radiotherapy [5,9,10,13,35] and the subsequent steps are dependent on correct gross-volume delineation. Field shaping and dose planning are based exclusively on the tumor volumes and critical normal tissue delineated. GIRAUD et al. (2002) suggested that the correct definition of the gross tumor volume can be attained, when radiotherapists are well trained in chest imaging [34]. SUNDAR and SYMONDS (2002) suggest a compulsory period of structured training in section imaging diagnosis for radiotherapists [36].

According to the recommendations of ICRU 50 (1991, 1993) and later on, of ICRU 62 (1999), gross tumor volume delineation should be performed as close as possible to the tumor and/or lymph node, without adding any safety margin [37-39]. Successive additional volumes are designated taking into account other treatment uncertainties. A second attitude adopted by the majority consists of attempting to distinguish between the tumor tissue and the surrounding collapsed parenchyma. This choice calls for perfect tomographic acquisition with rapid injection of the contrast medium and a first series of slices performed immediately after the injection [18].

Our results were incompatible with those of VAN DE STEENE et al. (1996), SENAN et al. (1999) and GIRAUD et al. (2002), which exhibited significant differences in lung tumor delineation [22,34,40].

In our study there was excellent intraclass correlation (Pearson's correlation coefficient) in the case of the radiotherapists and good correlation in the case of the radiologists. In the latter, the correlation was slightly lower due to a single point at which there was greater discrepancy between the first and the second measurement. In the analysis of radiotherapists and radiologists, we also observed excellent correlation between the two measurements.

Congruity was also evaluated by the Bland-Altman method, while randomness was observed in the distribution of data within the constructed confidence interval, and only one point fell outside the interval, indicating that the error among the measurements does not tend to increase when the measurement values are higher. Moreover, the average of the differences was close to zero, indicating good concordance between the two measurements.

The discrepant measurement of gross tumor volume of one of the radiologists resulted from associated atelectasis that constitutes the main cause of error in tumor volume delineation. We emphasize that in our study, we observed one case of tumor with mediastinal invasion, two with invasion of the thoracic wall and two causing lung atelectasis.

Some peculiarities of Hospital A. C. Camargo might have contributed to these results, such as integration of the radiotherapy and diagnostic imaging departments, internship of the radiotherapy residents in diagnostic imaging with learning of sectional anatomy, and geographical proximity of the radiology and radiotherapy departments, which are located in the same building, on adjacent floors.

Despite of there are no statistically significant differences in the definition of gross tumor volume between radiologists and radiotherapists in this study, in nine of twenty-three evaluated patients there was a difference greater than 20%, which can be clinically relevant. Most of these cases involved primary tumors located close to the mediastinum or chest wall, which hindered the proper measurement of the lesions. Regardless of the overlapping volumes have not been assessed, in neither case the observers considered different structures to delineate the target volumes.

Recently some authors have shown that delineation accuracy can be improved by using fluorodeoxyglucose-positron emission tomography (FDG-PET)/CT information. FDG-PET/CT is a functional study that has proved to be more accurate than CT in determining extent of non-small-cell lung cancer. Integration of FDG-PET/CT on the volume delineation can reduce interobserver variation compared with CT based delineation and alter gross tumor volume in about 50% of the cases. [41,42] FDG-PET/CT images are particularly useful in defining the target volume in the presence of atelectasis and in defining involved lymph nodes. [43]

## Conclusion

Radiotheraphy plays an important role in the management of inoperable lung cancer patients, A precise and consistent delineation of target volumes is needed to improve treatment and avoid complications. Although some authors have found large rates of interobserver variability on volume delineation for lung cancer, in this survey, there was no statistically significant difference in the definition of gross tumor volume between radiotherapists and radiologists or intraclasses. Some institutional characteristics should be responsible for this finding, such as integration between radiotherapy and diagnostic imaging departments.

## Competing interests

The authors declare that they have no competing interests.

## Authors' contributions

CJT conceived of the study, and participated in its design, acquisition, analysis and interpretation of data, and helped to draft the manuscript.

RC and PEN conceived of the study, and participated in its design and coordination, and helped to draft the manuscript.

PNVP, RCF and DGC conceived of the study, and participated in its design, acquisition of data, and helped to draft the manuscript.

MAB and AGVB have been involved in literature review, drafting the manuscript and revising it critically for publication.

All authors have given final approval of the version to be published.
